# Activities of stromal and immune cells in HPV-related cancers

**DOI:** 10.1186/s13046-018-0802-7

**Published:** 2018-07-05

**Authors:** Marconi Rego Barros, Cristiane Moutinho Lagos de Melo, Maria Luiza Carneiro Moura Gonçalves Rego Barros, Rita de Cássia Pereira de Lima, Antonio Carlos de Freitas, Aldo Venuti

**Affiliations:** 1 Laboratory of Molecular Studies and Experimental Therapy (LEMTE), Department of Genetics, Center of Biological Sciences, Federal University of Pernambuco, Cidade Universitária, Av. Prof Moraes Rego, 1235, Recife, PE CEP-50670-901 Brazil; 2 Laboratory of Immunological and Antitumor Analysis (LAIA), Department of Antibiotics, Center of Biological Sciences, Federal University of Pernambuco, Cidade Universitária, Av. Prof Artur de Sá, s/n, Recife, PE CEP-50740-525 Brazil; 3 Therapeutic Innovation Program, Center of Biological Sciences, Federal University of Pernambuco, Cidade Universitária, Av. Prof Moraes Rego, 1235, Recife, PE CEP-50670-901 Brazil; 4 HPV-Unit, Tumor Immunology and Immunotherapy Unit, Department of Research, Advanced Diagnostic and Technological Innovation, IRCCS Regina Elena National Cancer Institute, Via Elio Chianesi 53, 00144 Rome, Italy

**Keywords:** HPV-related cancer, Innate immune response, Cancer-associated fibroblasts (CAFs), Myeloid-derived suppressor cell (MDSC), Macrophages, Neutrophils, Natural killer cells, Immune evasion, Immunotherapy

## Abstract

The immune system is composed of immune as well as non-immune cells. As this system is a well-established component of human papillomavirus- (HPV)-related carcinogenesis, high risk human papillomavirus (hrHPV) prevents its routes and mechanisms in order to cause the persistence of infection. Among these mechanisms are those originated from stromal cells, which include the cancer-associated fibroblasts (CAFs), the myeloid-derived suppressor cells (MDSCs) and the host infected cells themselves, i.e. the keratinocytes. These types of cells play central role since they modulate immune cells activities to create a prosperous milieu for cancer development, and the knowledge how such interactions occur are essential for prognostic assessment and development of preventive and therapeutic approaches. Nevertheless, the precise mechanisms are not completely understood, and this lack of knowledge precluded the development of entirely efficient immunotherapeutic strategies for HPV-associated tumors. As a result, an intense work for attaining how host immune response works, and developing of effective therapies has been applied in the last decade. Based on this, this review aims to discuss the major mechanisms of immune and non-immune cells modulated by hrHPV and the potential and existing immunotherapies involving such mechanisms in HPV-related cancers. It is noticed that the combination of immunotherapies has been demonstrated to be essential for obtaining better results, especially because the possibility of increasing the modulating capacity of the HPV-tumor microenvironment has been shown to be central in strengthening the host immune system.

## Background

Innate immune response is critical for virus clearance. Its mechanism is essential for inducing an adaptive response, preventing infection chronicity and cancer development. HPV may cause the evasion of those mechanisms, which leads to host cell transformation [1]. Regarding this, immunotherapies aim to strengthen the host immune system to induce HPV clearance by means of the death of chronically infected cells and, thus, avoiding the spread of malignancy through the infected tissue. In head and neck squamous cell carcinoma (HNSCC), for example, low amounts of lymphocytes along with a decreased activity of natural killer (NK) cells and of antigen presentation are observed [2].

Recent therapeutic approaches have shown the great potential of the stimulation of innate immune mechanisms, generally increasing Th1 or cytotoxic cells activities at the final step or even, both [3]. Other studies focused on the inhibition of the viral oncoproteins activities [4], on the prevention of deflection in cytokines secretion towards Th2 profile and on the impairment of regulatory CD4^+^ CD25^+^ Foxp3^+^ T cells (Treg) [3].

A study field, which is receiving more attention currently, considers the importance of the cells adjacent to the HPV-related tumor and all the environment surrounding it, highlighting the stromal cells (e.g. fibroblasts) as well as the cytokines and chemokines they produce. These cells may be considered as co-factors for HPV-associated carcinogenesis due to the intense cross-talk between them and epithelial, tumor or immune cells at the tumor microenvironment [5].

It has been reported several times that cancer-associated fibroblasts (CAFs) are able to drive immunosuppression, cell growth and metastasis in HPV-related cancer as other (e.g. breast, fibrosarcoma, lung) [6]. This stromal cell, for example, was able to induce HPV16 immortalized keratinocytes penetration through Matrigel membranes by its conditioned media and the increased secretion of basic fibroblast growth factor (bFGF) [7]. Its counterpart, the normal fibroblasts, however, seem to restrain cancer development in favourable circumstances, as they could act as antigen presenting cells in a milieu with abundance of proinflammatory cytokines, such as IL-2 [8]. Therefore, the contribution of fibroblasts, CAFs and other stromal cells in HPV-related tumors is still poorly understood.

Instead of targeting fibroblasts, the cells of innate immune response have been more frequently evaluated and prioritized in novel immunotherapeutic strategies. They play a pivotal role by synthesizing cytokines, which attract other immune cells to the HPV-infection or -tumor microenvironment, by inducing T cell activation or differentiation and, in case of NK cells, by killing HPV-infected cells. Several studies have demonstrated the great potential of these cells in tumor prevention and eradication [9–11].

Other studies target macrophages, inflammation processes and the use of innate response adjuvants. Toll-like receptors (TLRs) ligands, cytokines and antibody-mediated blockade, such as anti-IL-10, anti-CTLA-4 (cytotoxic T cell-associated antigen 4), anti-PD-1 (programmed death-1 receptor), anti-PD-L1 (PD-1-ligand) and anti-TIM-3 (T cell immunoglobulin mucin 3) are fundamental tools also in HPV-related cancer treatment and prevention. All these tools may act synergistically with vaccines and with each other to increase their efficacy in cancer treatment [2, 3, 12, 13].

However, there is no immunotherapeutic approach capable of inducing cancer-free condition after the tumor has already established. In addition, the immune surveillance activities and how those can be used as weapons against HPV oncogenic activities are incompletely known. Therefore, this review aims to discuss the innate response elements and how they may be used in favour of infection resolution as well as prevention and treatment of the HPV-related cancers. In particular, it is highlighted the contribution of both cells with some immune properties (e.g. keratinocytes and stromal cells) and the immune cells themselves, such as the antigen presenting cells (APCs), natural killer cells, macrophages and neutrophils. Furthermore, a hypothesis of stromal cell-centered HPV-related carcinogenesis is presented.

## Keratinocytes

Keratinocytes (KC) are cytokine responsive cells able to play a role similar to the APCs’. Besides antigen presentation, they synthesize many signalling and regulatory molecules such as IFN-I (type I interferon), TNF-α (tumor necrosis factor-α), ICAM-1 (intercellular adhesion molecule-1), MCP-1)/CCL2 (monocyte chemoattractant protein-1/C-C chemokine ligand 2), MIP-3α/CCL20 (macrophage inflammatory protein-3α/C-C chemokine ligand 20), CXCL9 (C-X-C chemokine ligand 9) and antimicrobial peptides, which support activation and recruitment of immune cells [14].

**Fig. 1:**
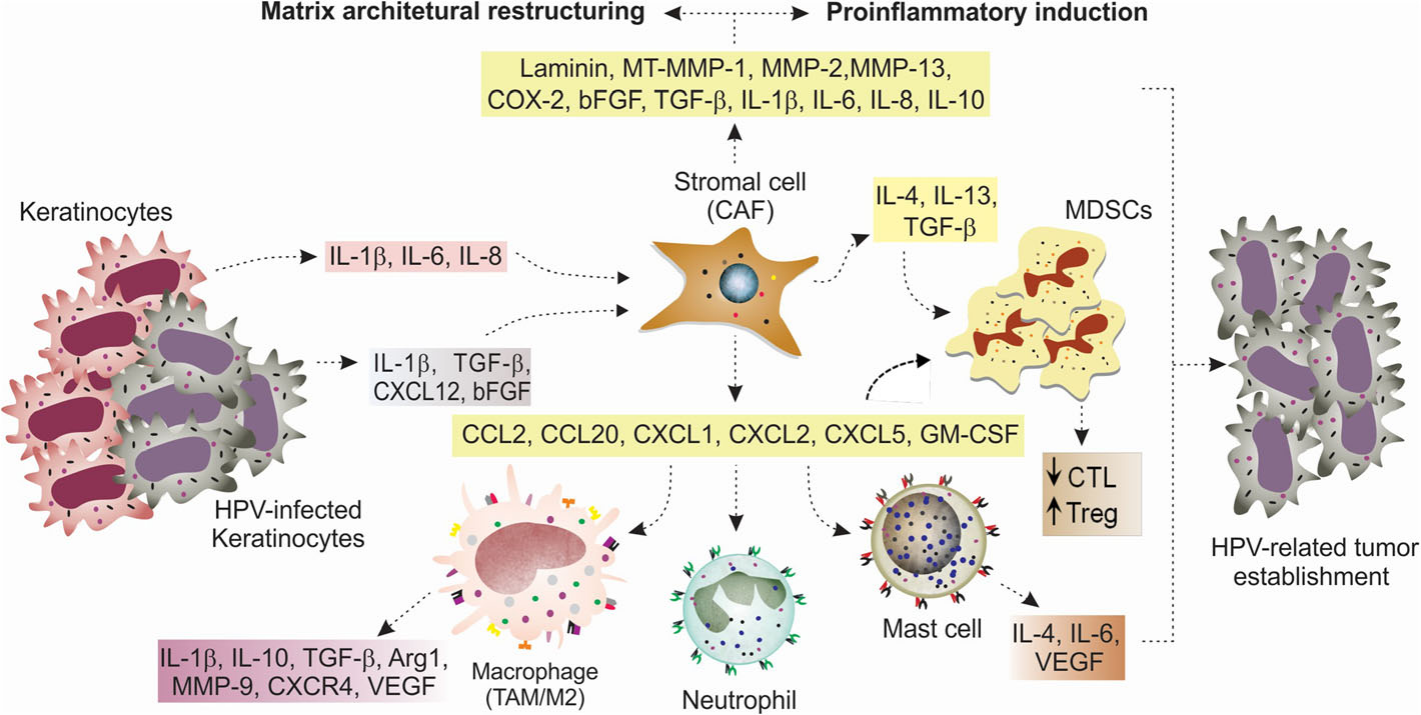
HPV-related carcinogenesis hypothesis involving stromal, immune cells and keratinocytes. Cells that show some alterations (dysplastic, hyperplastic) or immune cells induce stromal fibroblasts (by IL-1β) to secrete IL-1β, TGF-β and CCL20 in order to support infected cells to undergo transformation. HPV positive cells in intermediate and final degrees of neoplasia induce the phenotype change of fibroblasts to CAFs and they interact in a feedback loop to induce HPV-related carcinogenesis

Several studies have shown some viral immune evasion strategies involving the decreased activity of keratinocytes. Viral oncoproteins can disarrange the expression of many genes and transcription factors (e.g. NF-κB) in keratinocytes, including those modulating CTL activity, antigen presentation, cell communication, chemoattraction and pattern recognition receptors signalling (e.g. gangliosides synthesis on cell surface). Thus, molecules such as E-cadherin, TNF-α, IFN, IRF-1 (interferon regulatory factor 1), IL-6, IL-8, TLR9, TAP-1 (transporter antigen processing-1), CCL2, MIP-1α/CCL3 (macrophage inflammatory protein-1α/C-C chemokine ligand 3) and CCL20 are reduced [14, 15]. As consequence, balanced proinflammatory and chemotactic mechanisms are impaired and the presence of immune cells (e.g. APCs) turns compromised, leading to chronic infection β. An excessive and damaging proinflammatory response induced by keratinocytes, however, is able to support carcinogenesis by creating a pro-tumorigenic microenvironment. The increased synthesis of IL-1β [16], IL-6 and IL-8 can induce the expression of other proinflammatory genes including COX-2 (ciclo-oxigenase-2) and CCL20 by the adjacent fibroblasts and attract, among other inflammatory cells, tumor-associated macrophages (TAM) and Th17 cells (Fig. 1) [5]. These last cells are usually associated with tumor growth, angiogenesis and cancer development [17].

Immune approaches may be applied for KC modulation, mainly for inducing Th1 responses [15]. Several suggestive possibilities include: (1) stimulation of cytokine and chemokine production by the use of TLRs agonists; (2) stimulation of interaction between dendritic and NK cells which induce T CD4^+^ and CD8^+^ responses; (3) activation of keratinocytes ex vivo and re-implantation for production of proinflammatory and immunostimulatory molecules and presentation of antigens; (4) use of microRNAs or others epigenetic agents (e.g. 5-aza) to modulate the expression of key viral and human oncogenes in various cancer pathways; (5) administration of cytokines that turn keratinocytes and immune cells into the activated antitumor status.

## Stromal cells

HPV life cycle is greatly related to the stromal cells around it. Although still an underexplored area, it is known that these cells, especially fibroblasts, support virus interaction with epithelial cells as well as their infection by minimally exposing itself, which ensures the non-activation of host immune surveillance and disease progression [5]. The major role of stromal cells in HPV-related carcinogenesis, however, is to induce a proinflammatory milieu, a condition for establishment of HPV transformed cells. In addition, stromal cells support virus immune evasion, matrix architectural change, proliferation, invasion, angiogenesis and are associated with chronic infection [16, 18, 19]. Thus, this topic will discuss the importance of stromal cells (especially represented by fibroblasts and CAFs) in HPV-related carcinogenesis. In addition, a HPV-related carcinogenesis hypothesis involving stromal, immune cells and keratinocytes is presented.

In cervical cancer research, there are not a great number of studies involving fibroblasts effects on carcinogenesis, but they have made important contributions. In some occasions, it is difficult to do some evaluations since there are not specific markers for each fibroblast phenotype [20] and, as in a study where fibroblasts were marked by PDGFRα (Platelet derived growth factor receptor α), there is no clear differentiation between the normal and the cancer-associated phenotypes. From the studies evaluated, it is possible to describe some results:

i) Normal fibroblasts, induced by premalignant cells, were able to secrete specific growth factors, proinflammatory molecules and chemokines during the dysplastic and earliest stages of cancer generation [16];
ii) Normal fibroblasts had a positive effect on PDSC5-derived cervical tumor growth, showing an intermediate effect between PDSC5 administered with CAF and PDSC5 alone [16]. Besides, they enhanced the proliferation of HPV16 positive-cervical cancer cells (CSCC7 cell line) and the synthesis of TGF-β1 and MMP-7, important protumorigenic effects not showed by their counterparts with tumor-associated phenotype [19];
iii) CAFs were capable of causing proinflammatory effects and an intense stromal remodelling, even in the absence of cervical tumor cells or malignant transformation [16];
iv) Fibroblasts conversion to a proinflammatory signature, as demonstrated above, may be NF-κB-mediated and promoted by secretion of IL-1β by PDSC5 and dysplastic cells as well as macrophages [16];
v) It was found fibroblast proinflammatory signature in CIN I, III and carcinoma samples [16, 18];
vi) Normal fibroblasts were capable of inducing architectural changes in premalignant lesions, when malignancy were absence [5]. It is known fibroblasts brings epithelial-mesenchymal transition (EMT) through several ways: by augmenting the expression of metalloproteinases [5] or switching the FGFR2b/FGFR2c expression ratio by HPV16 E5 activity [21]. It is already known that HPV16 E5 is able to induce the expression of TGF-β and the switching of TGF-β signalling pathway from SMAD to PI3K/AKt, NK-kB and Ras/Raf. This results in  turning on the immunosuppressor [4] and probably, the EMT effects of TGF-β.

In HNSCC research, proinflammatory fibroblasts were also found commonly [22, 23], and IL-1β was demonstrated to play a key role in fibroblasts conversion to CAFs in an in vitro model [23], similarly as it was showed previously for cervical carcinogenesis. The growth factors secreted by fibroblasts also showed to be central for creation of a favourable milieu for cancer development, especially HGF (hepatocyte growth factor) and its signalling pathway through c-Met activation [24]. Interestingly, in a in vitro model of oropharyngeal carcinoma, HPV negative tumor cells induced normal fibroblasts to secrete a conditioned media, supporting tumor invasion and a positive evolutionary pressure so that cancer cells induced CAFs generation [22]. A parallel to this trait can be made for cervical cancer, although this fact should obviously be tested for this condition.

In summary, in both cervical and other HPV-related cancers, fibroblasts can support immune evasion and proinflammatory activities, as well as EMT, proliferation and invasion. They perform these activities by increasing: i) the expression of proinflammatory genes, including chemokines (CCL2, CXCL1, CXCL2, CXCL5, CCL20), COX-2, IL-1β, IL-6 and IL-8, ii) the recruitment of mast cells, macrophages and neutrophils, iii) the secretion of IL-10, TGF-β and iv) growth factors [5, 7, 16, 18, 19, 22] (Fig. 1).

Therefore, regarding the cited studies is possible to deduce that:

i) HPV-related tumor cells as well as HPV positive cells are not required so that fibroblasts can acquire proinflammatory/protumorigenic attributes; dysplastic and immune cells may play this role by secreting IL-1β and other key molecules which are almost completely unknown;
ii) Fibroblasts can be very useful for HPV-related carcinogenesis since the beginning and prior to the existence of neoplastic cells;
iii) The different fibroblast phenotypes may be both essential in HPV-related carcinogenesis and may assume complementary roles according to the function and time they perform their activities. The normal fibroblasts can be responsible for inducing inflammation, immune evasion and proliferation and thus, may be a part of an immediate support for dysplastic cells transformation in earlier stages of HPV-related carcinogenesis. On the other hand, CAFs may be responsible for keeping a prolonged stimulus for maintenance of cancer cells, and would be related to middle and later features of HPV-related carcinogenesis as EMT, migration and metastasis. Indeed, a study in cervical cancer correlated normal fibroblasts with cell proliferation and cancer-associated fibroblasts with cell migration [19].

**Fig. 2:**
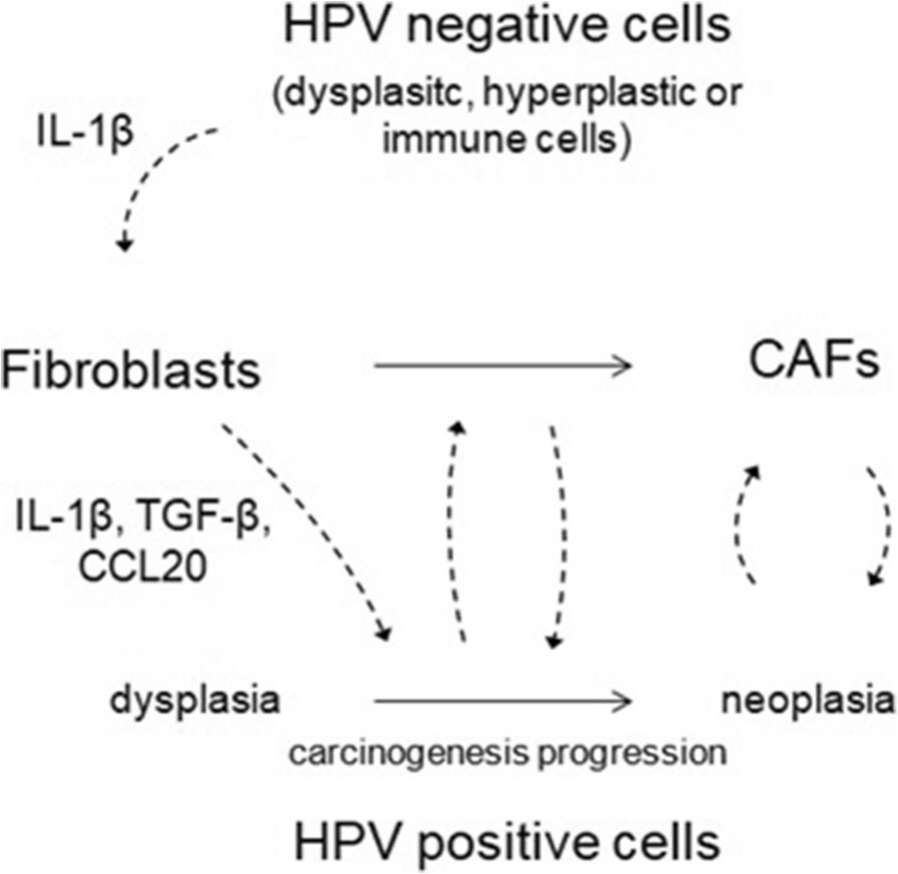
Stromal cells support HPV-related carcinogenesis through paracrine stimulation. Stromal cell plays a central role in HPV-related carcinogenesis. Cytokines and chemokines secreted by keratinocytes and HPV-infected cells (IL-1β, IL-6, IL-8, TGF-β, CXCL12 and bFGF) induce normal fibroblasts to become CAFs, which secrete a number of proinflammatory factors, chemoattractant and extracellular matrix remodelling molecules. This remodelling causes alteration of cancer cell polarity, migration, cell-cell communication and cell growth. The activation and attraction of immune inflammatory and modulatory cells, including M2 macrophages (TAM), neutrophils (TAN), mast and myeloid-derived suppressor cells (MDSCs) by key chemokines, further support the creation of a poor prognosis condition where T lymphocytes and NK cytotoxic responses are impaired, in contrast to the increased activity of Treg [5, 26, 27, 42, 43, 134]

Accordingly to what has been discussed, it was possible to draw a HPV-related carcinogenesis hypothesis involving stromal, immune cells, immunocomponents and keratinocytes (Fig. 2), and which is substantiated in the data presented previously in this section. It is possible to infer that HPV-related carcinogenesis may initiate with the interaction of dysplastic cells – not necessarily infected by HPV –, or even immune cells (e.g. macrophages) with normal fibroblasts, with the goal of supporting HPV positive cells to establish themselves as an immediate response from host stromal cells to the infection. Thus, during the prior and earliest stages of HPV-related carcinogenesis, the normal fibroblasts would secrete a set of proliferative, proinflammatory and immune evasive molecules, highlighting IL-1β, TGF-β and CCL20.

As fibroblasts create a prior favourable microenvironment for malignant condition, HPV positive cells gradually accumulate characteristics of neoplastic cells, among them the ability to cause the change of fibroblasts phenotype to CAFs. Once CAFs are created, they would support massively HPV positive cells on direction of transformation leading to cancer development and establishment, unlike what occurs to the HPV negative cells, which are not as much susceptible to CAFs conditioned media effects as the HPV positive cells are. The activity of CAFs, thus, might be a response of stromal cells to keep a prolonged stimulus for maintenance of cancer cells and would be related to later features of HPV-related carcinogenesis while normal fibroblasts would be related to the earlier ones. Therefore, like M1 and M2 macrophages performance on carcinogenesis (the first is associated with cancer onset while the second is associated with cancer maintenance and progression), normal and cancer-associated fibroblasts could be playing similar parallel roles respectively, in order to support cancer generation and progression like M1 and M2.

Normal fibroblasts, however, in other circumstances as in a milieu with a proper cytokine profile, can also play a role of containing carcinogenesis, as does M1 macrophages. In summary, the virtues of this hypothesis are especially five: one is that it seems fibroblast does not necessarily have to assume the cancer phenotype to produce protumorigenic effects, such as inflammation, architectural change, immune evasion and cell growth. Second is that fibroblasts are able to produce the cited effects prior to cell transformation at the earliest stages of carcinogenesis, when infected cells only show hyperplastic or dysplastic attributes. During this time, these cells secrete molecules which modulate fibroblasts to play protumorigenic role. Third and fourth are that it seems keratinocytes do not have to be infected or undergone transformation to induce phenotypic modifications on fibroblasts and finally, the fifth sets that normal and cancer-associated fibroblasts seem to have complementary roles and their activities are proper of specific carcinogenesis stages.

In conclusion, it could be seen that fibroblasts are quite important in HPV-related carcinogenesis, especially in cervical, and thus, studies on this issue are most frequently found (though not enough). Thus, the role in other HPV-related cancers could be underrated, which urges the existence of more studies about this subject as well.

## Macrophages and mast cells

Macrophage is a CD68^+^ CD86^+^ CD163^+^ monocyte-derived cell which plays an essential role for innate immune response and HPV elimination. These cells, upon activation by TLRs, LPS (lipopolysaccharide) and non-opsonic receptors, are able to phagocyte and secret pro-inflammatory cytokines and oxygen/nitrogen reactive species. They differentiate into two main phenotypes, the classic/inflammatory M1 (CD86^+^) and the alternatively activated M2 (CD163^+^ CD206^+^), also known as tumor associated macrophages. Some authors consider TAM as a third phenotype with characteristics of both, but primarily of M2 [25].

These cell subtypes show different but complementary roles in HPV carcinogenesis, which can be correlated with the specific inflammation level they are associated with. Since inflammation is a crucial step for HPV clearance as well as for cancer generation and thus, can assume opposite effects according to the intensity or the moment it occurs during carcinogenesis, the control of inflammation through regulation of TAM is a interesting therapeutic target for HPV-related cancers.

Scientific data correlate macrophages, especially M2 or TAM subtypes, with tumor progression and low overall survival in both cervical [26] and other HPV-related cancers [27]. These cell phenotypes have a negative impact on cervical cancer therapy [17] and induce tumor growth, angiogenesis, metastasis and immunosuppression by:

i) Boosting Th2 response through IL-4, IL-10 and IL-13 synthesis [18];
ii) Inhibiting CD4^+^ and CD8^+^ T cells responses [17];
iii) Secretion of the vascular endothelial growth factor (VEGF) and other proangiogenic factors [5, 17].

It was reported that poor prognosis is linked to increased levels of CD163 (TAM marker with M2 phenotype), CSF-1 (colony-stimulating factor-1), major regulator of macrophage lineage, and Arginase-1 (Arg-1) – markers of TAM’s activity – in several cancers including the HPV-related ones [12, 25, 28]. Increased infiltration of CD163 macrophages in stromal and peri-tumoral areas were associated with malignancy progression, lymph node metastasis, higher FIGO stages [29], higher lesion grades and cervical cancer specimens [28, 30].

In HNSCC, high levels of TAM were found in tumor microenvironment and were associated with CTLA-4-related immunosuppression, the expression of PD-L1 and immunosuppressive cytokines, as well as metastasis and poor prognosis [27]. In oral squamous cell carcinoma (OSCC), TAMs were recruited by IL-1β-mediated upregulation of CXCR4 and CXCL1 and were associated with tumor migration, invasion and angiogenesis [31]. CD68 and CD163 (TAM markers) were observed to be correlated with lymph node status and with several cancer stem cell markers such as SOX2 (sex determining region Y) and ALDH1 (aldehyde dehydrogenase 1). These markers and CD163 were also associated with poor overall survival [32], cancer invasion and worse clinical outcome [33].

However, macrophages can also contribute to lesion regression, inducing tumor cell lysis and carcinogenesis interruption, especially the M1 subtype, which synthesizesTNF and NO (nitric oxide) [12]. M2 is associated with Th2 response, while M1 induces Th1, which favors HPV clearance. In a study correlating M1 and M2 levels in advanced cervical cancer treated with cisplatin-based CT/RT followed by radical surgery, patients with higher M1/M2 ratio showed longer disease-free survival and a complete pathologic response [34]. The virus itself hampers host immune responses, regulating M1 activation and differentiation. It downregulates MCP-1 and MIP-3α [35] expression, regardless of inducing the decrease of IFN-γ levels (this IFN activates M1) and the increase of TGF-β (able to activate M2) [17]. Polarization of macrophages to M2 subtype due to deficiency of IL-12, IL-18 and GM-CSF and the raise of CCL17, CCL22, IL-4, IL-10, IL-13 and CSF-1, favours tumor establishment through the prevention of cytotoxic T response and stimulation of immunosuppression [25].

Targeting macrophage polarization to the appropriate phenotype, associated with increased Th1 and cytotoxic responses and blockade of COX-2-mediated differentiation to M2 profile [36], proved to be good options for cancer therapy or prevention [25, 35]. Other approaches are: blockade of chemoattractants (e.g. anti-CSF [27, 37, 38], anti-VEGF, anti-CXCL12 [27]), signalling pathways (e.g. NF-κB) and cytokines secretion [31]; and depletion of TAMs from the tumor milieu [39].

Mast cell is another important effector cell for development of the innate immune response and may have its importance in carcinogenesis. These cells have secretory granules which contain large amounts of proteases. They were found in CIN2 and CIN3 stages, but their role in HPV lesions is uncertain. Mast cells may act in an immunoprotective or immunosuppressive way. In the first case, the secretion of proinflammatory cytokines recruits cells of the innate and adaptive immunity inducing apoptosis of tumor cells [12]. Mast cells can be used as an enhancer of antibody responses, upon stimulation with CTA1-DD/IgG. This compound can be considered as an adjuvant, increasing the effectiveness of HPV16 L1 VLP vaccination with poor to none toxicity [40]. On the other hand, mast cells can induce immunosuppression by secreting IL-10 and VEGF that support immune modulation and inflammation [12]. In a model of E7 transgenic K14 mice, it was shown that mast cells were attracted by E7 expressing cells in a CCL2/CCL5-dependent manner and induced immunosuppression by preventing T CD8^+^ response [41].

## Myeloid-derived suppressor cells and HPV-Tumor cells

MDSCs compose a population of immature cells HLA-DR^−/lo^ Gr-1^+^ CD11b^+^ CD14^−^CD15^+^ CD33^+^ for polymorphonuclear-MDSC, or HLA-DR^−/lo^ Gr-1^+^ CD11b^+^ CD14^+^ CD15^−^ for monocytic-MDSC. They belong to myeloid lineage and are lumped during chronic inflammation and cancer, forming a heterogeneous population with immunosuppressive role [42, 43].

**Fig. 3:**
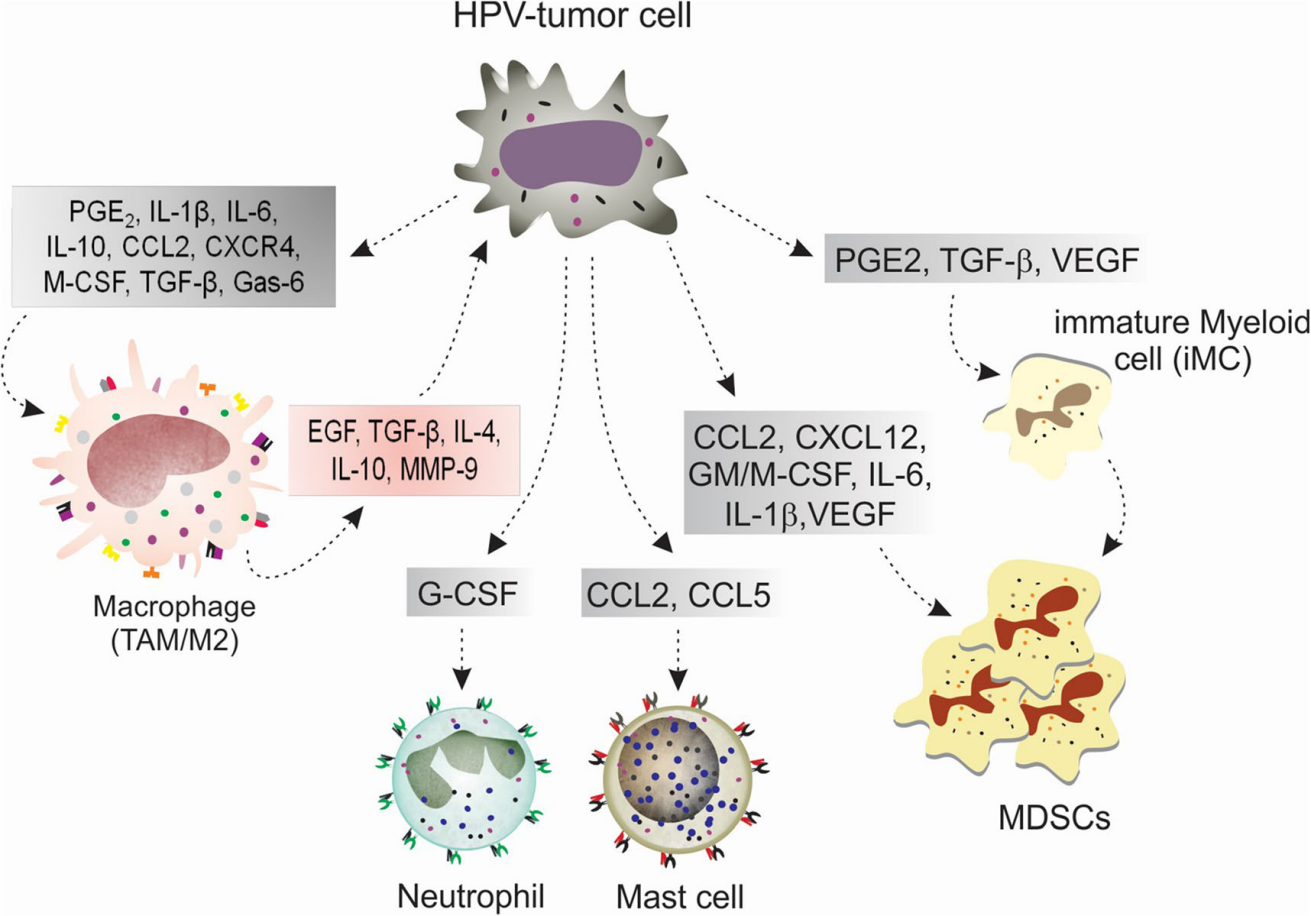
Cross-talk between tumor, MDSCs and immune cells for development of HPV-related carcinogenesis. As demonstrated in the figure and in the text, HPV-tumor cell plays a central role in HPV-related carcinogenesis. This cell is able to secret a number of proinflammatory and immunosuppressive factors, which induce macrophage differentiation towards M2 phenotype, iMC differentiation towards MDSCs as well as the recruitment and activation of both MDSCs and proinflammatory immune cells (macrophages, neutrophils and mast cells). Major molecules for the occurrence of these events are: CCL2 and CXCL12, for cell recruitment; TGF-β, IL-1β and IL-10, for immunosuppression; MMP-9, for tumor invasion; and STAT3, due to the activation of JAK/STAT3 intracellular signalling pathway which enables the occurrence of cell response to all stimuli caused by the engagement of the shown cytokines and factors to their respective receptors

In cancer study, including cervical and other HPV-related cancers, MDSCs are  usually associated with both poor clinical outcome and with therapy resistance. They are activated and recruited by several inflammatory mediators secreted by stromal (Fig. 1) and tumor cells (Fig. 3) in cancer milieu in order to support inflammation, immunosuppression, tumor invasion and angiogenesis. They prevent CTLs activities, downregulate IFN-γ and induce the augment of Treg levels, supporting carcinogenesis. In addition, their levels were augmented when compared with normal controls, showing directed relation with pathological grade [42–47].

In cervical cancer, it was observed that tumor cell induced MDSCs proliferation and their accumulation in lymphoid organs [44]. These cells were capable of suppressing CD8^+^ T responses by production of NO and MHC I molecules [45], the latter probably by the activation of the inhibitory signalling pathway. In HNSCC, MDSCs were also found with elevated levels mediated by chemoattractants (e.g. CXCL1, CXCL5), which were synthesized by NF-κB induction. It was also observed MDSCs induced angiogenesis, inflammation and immunosuppression by the reduction of CTLs activities and upregulation of Treg cells [47].

Due to MDSC role in HPV-related carcinogenesis, several therapeutic approaches aim to inhibit MDSC activity in order to reverse immunosuppression, improve host immune response and thus, support HPV clearance [12, 48]. In a C57BL/6 mice model, all-trans retinoic acid combined with a fusion protein (E6 + E7) vaccine reduced MDSC levels, enhanced antitumor response (by increasing DC levels and CTL cytolytic activity) and improved survival [48]. In HNSCC, tadalafil is being tested in ongoing clinical trials in order to downregulate iNOS and Arg-1 activities in MDSCC and induce antitumor responses, as demonstrated in mice model. Other immune approaches, in order to suppress MDSC functions and/or recruitment, are also being tested in HNSCC treatment, such as the depletion of MDSC along with anti-CTLA-4 monoclonal antibody (in order to enhance anti-CTLA-4 antitumor effects) [49], the use of STAT3 (AZD9150), and the recruitment of inhibitors (e.g. MJ18, a B7-H3 antagonist; AZD5069, a CXCR2 antagonist) [50, 51]. CXCR2 is a key receptor since its ligands are CCL2 and CCL5, which are elevated in tumor milieu and recruit, in addition to MDSCC, M2 macrophages, neutrophils and mast cells, all the cells related to a harmful inflammatory response and poor prognosis.

The HPV-tumor cell (Fig. 3) plays a central role in carcinogenesis. This cell is able to synthesize costimulatory and chemoattractant mediators that induce immunosuppression and prevent host immune surveillance through the interaction with immune cells. Due to this, tumor cells are great targets for immunotherapeutic approaches. These cells can be modified in vitro to express genes encoding cytokines to enhance immune response, such as IL-2, IL-12 and GM-CSF [52]. Also, peptides from HLA molecules can be recovered or predicted in a reverse immunological approach and tested for APC activation and T cell sensitization for target cells; antigens can be (i) found from the cross test of cDNA libraries (from tumor cells) with autologous serum, or (ii) transfected in target cells with appropriate HLA for evaluation of T cell stimulation, or (iii) discovered by microarray or gene expression analysis for noticing of highly expressed genes and proteins from tumor cells [53].

### Neutrophils

Neutrophils are innate immune cells derived from granulocyte-monocyte progenitor lineage and responsible for phagocytosis and secretion of reactive oxygen species. They are abundantly localized in circulation and are suggested to play a central role in preventing immune responses in advanced cancer patients. Neutrophils support inflammation by secreting central inflammation mediators in infected or injured areas, such as the pro-angiogenic and proteolytic enzyme MMP-9 [54].

Few studies have reported about neutrophils and cervical or HPV-related cancers and thus, its role is uncertain. In cervical cancer, neutrophils were found in large amounts in high grade and cervical cancer lesions [55]. As observed in other tumors [54]. these cells are usually associated with poor prognosis [56], being considered as an independent factor for short recurrence-free survival [57], as well as the neutrophil-to-lymphocyte ratio (NLR), which was associated with unfavourable overall and progression-free survivals. NLR was also associated with a large tumor size, advanced clinical stage and lymph node metastasis [58]. Therefore, neutrophils are also called CD66b^+^ tumor-associated neutrophils (TAN).

In HNSCC, TAN were also frequently found with elevated levels and NLR was associated with poor prognosis [59, 60]. High ratios were reported as a poor prognostic factor, decreasing overall survival in a largest patient study (*n* = 543) [61]. This ratio is also useful in differentiating laryngeal squamous carcinoma from benign laryngeal lesions. However, the relationship between NLR and clinical outcomes seems to be dependent on HPV status in HNSCC. In HPV positive specimens NLR was lower than in HPV negative samples, and when the HPV status was taken into account the association between NLR and survival was not statistically significant [62]. Similarly, the increased levels of neutrophils were associated with poor overall and recurrence-free survivals only in HPV positive oropharyngeal cancer patients [63].

## Dendritic cells and immune checkpoint molecules

**Fig. 4:**
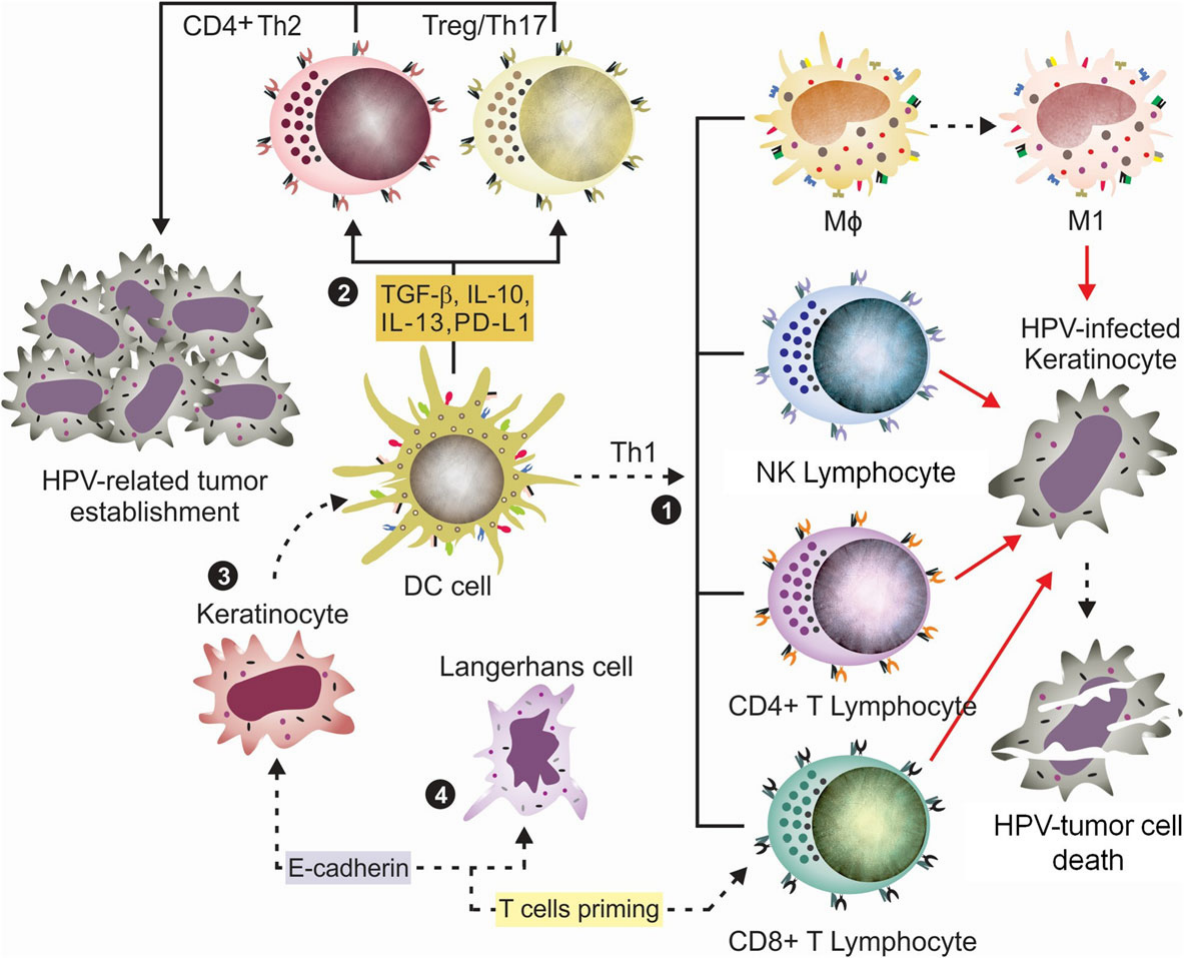
Central role of dendritic cell in host immune response against virus. (1) Under the stimuli of TNF-α, IFN-γ and other molecules, DCs undergo maturation and produces type I IFN, IL-12, GM-CSF, B7 molecules (CD80 e CD86) and LFA-3 (lymphocyte function-associated antigen 3). IFN I causes NK cell activation; IL-12 and -18 promote Th1 and M1 cell profile differentiation; GM-CSF stimulates granulocyte and monocyte production and function; B7 and LFA-3 are co-stimulatory molecules of T cell in increasing inflammatory response. The Th1 profile is able to induce tumor death. (2) When DC are partially or completely immature, they play an immunosuppressive role, inducing Th2 and Treg differentiation by secreting immunomodulatory cytokines, such as TGF-β, IL-1, − 6, − 10, − 13, 23 and PD-L1. Th2 profile promotes T cell anergy and tumor establishment. (3) Similar to NK and CD8+ T cells, keratinocytes can activate DC through CD40L. (4) E-cadherin is the protein that promotes cellular interaction between LC and KC. In HPV infected tissues, E-cadherin is downregulated by the E6 oncoprotein. LCs can be activated by heparan sulfates, PAMPs, IFN-γ, TNF-α and IL-17 in order to secrete a large range of immunoprotective molecules (IFN, TNF-α, ICAM-1, AMPs, IL-1, − 6, − 10, − 18, CD40L, CCL20, CXCL9, − 10) [1, 14]

Mature dendritic cells are those positive for CD11c, CD40, CD80, CD83, CD86, CD209 and HLA-DR markers, and are essential in immune response against HPV. Due to its plasticity and the presence of multiple receptors on its surface, DC can interact with all cells of the immune system [64] and thus, are critical for initiation of antiviral and antigen-specific immune responses (Fig. 4). The maturation status of this cell is essential for an effective immune response against HPV and thus, DC has been used in several different immunotherapeutic approaches [3, 12, 65].

The occurrence and development of HPV-associated cancers are related to changes in the levels, activity or maturation of dendritic cells. Several studies have found association between the malignancy progression and the downregulation of co-stimulatory markers, such as CD40, CD80 and CD86, as well as the increased expression of inhibitory markers, such as PD-1, PD-L1 and CTLA-4 [2, 14]. It was seen, for example, that cervical cancer cells may favor tumor establishment by inhibiting the migration of DCs to lymph nodes (through depletion of CCR7), and inducing DC to produce MMP-9 [66].

CD40/CD40L interaction is a central signalling pathway for dendritic maturation and for inducing CD4^+^ and CD8^+^ responses during HPV chronic infection [67]. In a HeLa model of cervical carcinoma study, , this interaction was able to repress transcriptional activity of HVP18-P105 promoter and increase IL-6 synthesis [68]. Thus, therapeutic approaches were developed/tested (e.g. clinicaltrials.gov identifier NCT00319748) or are under clinical trials (e.g. NCT03418480) to induce the activation of this pathway aiming the prevention of hrHPV genes expression and promotion of higher CD4^+^ and CD8^+^ responses [68, 69].

The PD-L1 marker on DCs is an important molecule for modulation of Treg and T CD8^+^ cells activities, constituting an essential immune HPV-escape pathway. The DC-PD-L1^+^ cell subpopulations are more abundant in hrHPV^+^ patients and are correlated with the downregulation of Th1-type cytokines. In this condition, T cells undergo a functional exhaustion and anergy through the activation of the PD-1/PD-L1 inhibitory pathway. In cervical cancer [70] as well as in HNSCC [2], PD-L1 was found upregulated. PD-1/PD-L1 axis activation causes a loss of function of CTL cells which can be restored by the administration of antibodies that blocked PD-1/PDL-1 interaction [14]. In oropharyngeal and other head and neck cancers, PD-1/PD-L1 pathway has been suggested to be a major mechanism of immune escape [14].

In addition to DC and tumor cells, PD-L1 was also found on the surface of stromal cells and M2 macrophages, markers of poor prognosis as discussed in previous sections. As a consequence, PD-1/PD-L1 axis was eventually correlated with a reduced disease survival rate and was suggested as a promising immunotherapeutic target alone or in combination with other therapeutic approaches, such as the administration of EGFR (endothelial growth factor receptor) or CTLA-4 antagonists – cetuximab and ipilimumab, respectively. The anti-PD-L1 antibodies durvalumab (NCT02207530) and atezolizumab (NCT03073525), and the anti-PD-1 antibodies pembrolizumab (NCT02291055, NCT02255097 and NCT02252042) and nivolumab (NCT02054806, NCT02105636 and NCT02488759) have been tested in clinical trials (phases I and II) for both cervical and head and neck cancers [2, 13].

The CTLA-4 has also demonstrated an inhibitory activity on DC activation of cytotoxic T cell responses [14]. This inhibitory receptor belongs to the B7 receptor family, being expressed by Treg cells and binds to CD80 and CD86 ligands on DC cells surface with higher affinity of CD28. As in cervical cancer as in HNSCC, CTLA-4 was found to be highly expressed, being frequently associated with poor prognosis [2, 14]. Anti-CTLA-4 monoclonal antibodies or CTLA-4 vaccines constructions were developed in order to prevent CTLA-4 activities and it has been tested or are in ongoing trials for treatment of cervical cancer [14, 71, 72] and HNSCC [2, 13].

Another immune checkpoint protein, which is recently gaining more attention, is TIM-3. In a transgenic HNSCC mouse model, this protein was associated with an augmented Foxp3, CD68 and CD163 levels, markers of Treg and macrophages respectively, and with an increase of Treg and M2 (CD11b^+^ CD206^+^) cells. A decrease in tumor growth and in Treg and CTLA-4 levels was observed following TIM-3 blockade, while M2 macrophage markers were not altered. Also, TIM-3 blockade caused a significant increase in IFN-γ synthesis, which highlights the importance of the use of this immune checkpoint protein in possible future immunotherapeutic approaches [73].

Although mature dendritic cells have been associated with viral immune-protection, it was seen that immature DCs are upregulated by hrHPV and can cause host immunosuppression and immunotolerance in cervical cancer studies. These properties may be related to HPV-mediated increased levels of IL-10 and TGF-β derived from cervical cancer and Treg cells. Immature DC can induce Treg cell differentiation by secreting IL-10 via a feedback mechanism. This DC population is associated with high grade and cervical cancer lesions, and is involved in local immunosuppressive activity. In a in vitro, it was reported that the ineffective HPV16-E7 graft rejection in immunocompetent syngeneic recipient mice was characterized by the lack of costimulatory molecules and the expression of immunoregulatory ones (e.g. indoleamine 2,3-dioxygenase) on DC surface [14].

In summary, the control of the immune response by DC depends on precise timing of existing cytokines at tumor milieu. These molecules promote the necessary signals for accomplishment of DC full maturation and the suitable subset differentiation (immune effector versus immunosuppressive). Each subset has its specific cytokine profile, which configures the immune activity that ensures the appropriate host immune response.

### Dendritic cell vaccines

Several promising therapeutic approaches for cervical and other HPV-associated cancers have been focused on activation of DCs. The goal of these methods was to enhance DC antigen presentation in order to increase viral immunogenicity and, consequently, the induction of Th1 and/or CTL responses. The most frequent high risk type, HPV16, has been widely utilized as pathogen model [65, 67, 74, 75].

Studies reported pre-clinical and clinical trials based on dendritic cell vaccines for cervical cancer and HNSCC [75–77]. DCs are very cytotoxic towards HPV16 E6 and E7 proteins expressing cells [78], thus autologous DCs are generally pulsed with these antigens (E6, E7 or both peptides or proteins), with ex vivo altered tumor cells [53, 75, 76] or with transfected DNA to be activated [53]. Both oncoproteins (E6 and E7) are the closest to ideal tumor associated antigens [53]. However, clinical effects are generally missing despite the strong immunological responses. Therefore, adjuvants are usually used in DC-based vaccine approaches in order to potentiate T cell activation and cytokine secretion, like IL-2, TLRs ligands (i.e. CPG, LPS, poly(I:C)) or other antigens [75, 79, 80]. An interesting study, for example, evaluated in vitro and in vivo the augment of antitumor responses (was seen an upregulation of IFN-γ and IL-12 levels and CTL activities) and the decrease of cervical tumor size [79]. Another study demonstrated an augmented immune response against cervical preneoplastic cells by using women-derived DC pulsed with HPV16 E7 oncoprotein with cholera toxin [81].

The use of adjuvants is also common and required in other vaccine approaches, such as protein- or peptide-based [53]. These adjuvants aim to pulse DC activity, e.g. poly(I:C) and anti-CD40 monoclonal antibody. When both adjuvants were administrated with a HPV16 E7-peptide vaccine, they caused the clearance of HPV16 E7 tumor cells in mice [3]. CD40 showed the best activity (together with LOX-1 and Dectin-1) among DC surface receptors [67, 82] inducing protection in mice with HPV16 E7-expressing tumor [75]. In other example, an HPV16 E6 peptide vaccine showed a significant increase in Th1 response and regression rate (83%) when used together with Candida skin reagent [83]. Currently, a peptide-based HPV16 vaccine using MAGE-A3 is being tested combined with GM-CSF and montanide ISA-51 (an adjuvant of immune response) for treatment of HNSCC (NCT00257738). GM-CSF has shown to be a promising cytokine for immune induction [53].

Furthermore, DC can be transduced (e.g. through adenovirus), encoding the specific antigen of interest. The main advantage of this approach is the prevention of HLA restriction boosting antigen presentation [52]. DC can also be transfected with siRNA targeting key cytokines, such as IL-10 [67, 75]. Currently, the use of pre-immature dendritic cells is alternative to DC for being less laborious and having shorter production time, without the need of using IL-4 in culture medium for DC maturation [52].

### Langerhans cells

Langerhans cells (LCs) are a subset of myeloid DC (CD11c^+^) with specific markers as CD1a^high^, CD11c^interm^, langerin (CD207), E-cadherin^+^ and EpCAM (epithelial cell adhesion molecule) [1, 14]. They are located in epidermis and mucosa and are the first cells responsible for presenting viral antigens owing to the infection proximity, but they are not able to induce a sufficient T cell immune response because of an inappropriate costimulatory microenvironment [35]. Some studies reported the reduction in the number of LCs and in the expression of adhesion and costimulatory molecules by these cells in CIN lesions [14], suggesting that HPV could prevent its recognition by a restricted upregulation of LC key genes [84]. It is known that hrHPV oncoproteins can modulate both Langherhans and dendritic cells activities, which leads to the inhibition of immune surveillance (Table 1).

**Table 1:** Summary of oncoproteins immune evasion mechanisms on LCs and DCs

Oncoprotein	Action-mechanism
E5	Downregulates MHC I and II classes and impairs the transport of these molecules to cell surface [4]
E6	Reduces the number of intraepithelial LCs in CIN lesions accordingly to grade severity [131]
	Reduces the expression of E-cadherin in infected keratinocytes and in higher lesion grades, disrupting adhesion of keratinocytes to LCs [14]
	Impairs monocytes differentiation to Langerhans cells in vitro [135]
	Induces death of precursor or differentiated monocytes in vitro [135]
E7	Downregulates essential molecules involved in antigen processing and presentation, such as TAP-1, TAP-2, LMP-2, LMP-7 and MHC I components [1]
	Impairs gene expression of adhesion markers and costimulatory molecules in LCs [14]
	Shifts cytokines profile: reduces TNF-α expression and induces IL-10 synthesis [14]
	Along with E6, impairs APCs recruitment to infected sites because it reduces the expression of chemoattractant molecules such as IL-8, CCL2 and CCL20 [14]

LCs levels were also found decreased in the stroma of oropharyngeal cancer patients with HPV infection [14], an outcome which could be related to downregulation of CCL20 and E-cadherin [14]. A recent study pointed out higher levels of LCs in stromal compartment as strong prognostic markers of recurrence-free and overall survival in HPV-negative HNSCC patients. It was also found that the number of LCs was significantly lower in HPV-positive patients than in HPV-negative ones [85], what could be related to viral oncogenes activities on LCs. In laryngeal squamous cell carcinoma, LCs infiltration was related to longer disease-free survival, lower local recurrence and less lymph node metastasis [86]. Similarly, in tongue carcinoma patients, higher number of CD1a-positive LCs around the tumor was associated with decreased recurrence and better survival rate [87].

However, the amounts of LCs in HNSCC were variable and controversial in several studies. Some studies showed that LCs were decreased in OSCC [88], but increased in HNSCC patients, even though an increase in LC number was correlated with a recurrence-free survival [14]. In another study, LC was found decreased in lip cancer when compared with the oral cavity squamous cell carcinoma [89].

## NK and NKT cells

Natural killer cell is defined as innate lymphocyte CD19^−^ CD14^−^ CD3^−^ CD16^+^ CD56^+^ CD69^+^ NKp46^+^, which plays key role in antiviral and antitumor immune responses in HPV-related cancers. In addition to its cytotoxicity role, it can secrete a large amount and variety of signalling molecules directed by the cytokine pattern within the tumor milieu. As a consequence, NK cell is able to regulate several others cells, such as lymphocytes, macrophages, dendritic, stromal and endothelial [10, 90].

Since NK cell plays a central role in immune response against HPV, causing viral clearance and cancer prevention, this cell has been frequently assessed in HPV-related carcinogenesis, especially the cervical. It is known that during the course of common cervical HPV infection, i.e., when infection is resolved, NK cell is activated by KC and DC (this latter through a close interaction). Following, it becomes capable of performing a cytotoxic response, as well as T cell priming and maturation, which leads to the elimination of HPV-infected cells [4].

### NK cells in cervical cancer

In cervical cancer, otherwise, NK cell activities and the susceptibility of infected and cancer cells to NK cell effects have been prevented by HPV oncoproteins. It was observed, for example, that HPV16 E6 and E7 prevented type I IFN signalling, the synthesis of IFN-γ induced by IL-18 [91] and the expression of MHC class I [92], and CXCL14 (the latter two by HPV16 E7) [93] to create an adverse microenvironment to NK cell cytotoxic activity. As well, it was observed the reduction of NK count and activitiy in preneoplastic [94, 95] and cervical lesions with an active HPV 16 neoplastic infection [91, 96]. Likewise, an increased number of NK cells was also found in precancerous lesions when compared with normal samples by flow cytometry; this found might be related to a host response against infection [97]. As NK cells activities are associated with lesion regression, the observation of disease outcome would be interesting information for explaining the result.

As NK cell activities depend on the receptors and ligands associated to this cell, several studies evaluated the effects of these molecules on cervical carcinogenesis. It was suggested that cervical cancer cells could induce the reduction of NKG2D and NKp46 levels on the NK cell surface and this was correlated with the reduction of cytotoxic activity [98]. NKG2D downregulation was also seen in patients with cervical cancer by Treg cell activity [99], which might be related to the induction of TGF-β synthesis [10, 100] and the inhibition of IFN-γ secretion. Moreover, the patients with cervical cancer or with high-grade lesions have shown a reduced expression of NKG2D, NKp30 and NKp46 [94, 95], the same receptors which were found with increased levels after the administration of the quadrivalent [101] and bivalent (NKG2D) [102] vaccines and supported host immune response. Regarding NKG2A, at least to the best of our knowledge, only two studies reported an increased expression in cervical cancer patients and it was not on NK cells, but on CTLs [103, 104].

Other receptors have also been evaluated in cervical cancer such as the killer cell immunoglobulin-like receptors (KIR) class. This class of molecules includes a large range of highly variable receptors with opposite functions (the majority shows NK cell inhibitory signalling activity) [105]. In a cohort study with western Australian patients, no significant association was observed between any evaluated KIR and high-grade lesion or HPV16 or 18 genotypes [106]. In a elder and similar study, however, (KIR)3DS1, an activation receptor, which has been associated with a good prognosis in HIV infection due to the significant increase in antiviral immune activity, was associated with cervical neoplastic progression to carcinoma [107].

Curiously in this study, (KIR)2DS1 and (KIR)2DS5 – which are activation receptors as well –, were also more frequently found in CIN3 and cancer samples (compared with normal controls and as occurred to (KIR)3DS1); although their *p* values were not significant, they were very close (0.066 and 0.078 respectively). In this study, increased risk of cervical cancer development was associated with a stronger activated phenotype in a gradual spectrum of KIR-related NK cell activation (with the presence of NK receptors and their ligands) [107]. Probably, by the attempt to turn NK cells activated, host immune system tries to counter the progression of malignant cells. Interestingly, the (KIR)3D receptors recognise HLA-A and HLA-B [108], the same types which HPV16E5 specifically induce downregulation to prevent NK cell activation [4]. The combination of KIR (genes) and their ligands (HLA) have not been evaluated yet regarding the relapse rate in cervical carcinogenesis as had been performed in other diseases [109].

NK cell ligand levels are also important for an appropriate immune surveillance in cervical cancer. A study revealed an increased expression of HLA-E associated with the absence of NK cells at tumor milieu [104] and other study reported the downregulation of HLA-E by HPV E7 induced-methylation in human keratinocytes [110]. In ovarian tumors HLA-E was associated with a frequent expression of CD94/NKG2A in CD8^+^ T cells. Another MHC subtype, HLA-G, was reported to be involved in the cervical carcinogenesis as well. This ligand might play its activities indirectly by the presence of HLA-E and several haplotypes were correlated with high-grade lesions [111]. In addition, this ligand interacts with the NK receptors and causes the suppression of cytotoxic activity inducing the apoptosis of NK cell and the upregulation of inhibitory receptors [112].

HLA-Cw group 1, in its turn, was observed to be significantly overtransmitted in women with invasive cervical cancer, especially in the women infected by HPV16 or 18 [113], while HLA-Cw group 2 was associated with a decreased risk of cervical cancer development [107]. As these molecules bind to (KIR)2DL inhibitory receptors, another studies also evaluated the association of several HLA-C/KIR combinations levels with cervical cancer risk [114, 115], confirming the importance of these molecules interaction in cervical carcinogenesis through the modulation of NK activation/inhibition balance.

Other NK ligands extensively studied in cervical cancer research have been MICA (MHC I polypeptide-related A chain) and MICB (MHC I polypeptide-related B chain) – both interact with NKG2D. These ligands, on the surface of cervical tumor cells, boost cytotoxic response against the malignant cells by the engagement with receptors on NK cell and CTL, and thus, were related to good prognosis [116] and suggested as potential immunotherapeutic tools [117, 118]. The soluble or secreted forms of these ligands (sMICA and sMICB) were found augmented in serum of patients with cervical and precancerous lesions when compared with healthy donors (sMICA) [94], in cervical cancer lines [119] and were associated with poor prognosis [118]. Both ligands induced a downregulation of NKG2D expression [94, 98] and this is suggested to be an immune evasion mechanism performed by hrHPV to lead to cancer development [120], since the engagement of NKG2D and MICA/MICB plays an important role in cervical and other cancer immune surveillance [95, 98, 117, 118, 121]. Altogether, these studies reveal that modulation of NK cell receptors and ligands affect immune response against HPV.

### NK cells in other HPV-related cancers

Although fewer studies have been conducted on HNSCC compared with cervical cancer research, consonant results were reported. It was observed a decreased number and impaired activity of NK cells in mouse and in patients, as well as increased rate of spontaneous apoptosis [100]. In contrast, a recent clinical trial verified that HNSCC has one of the highest NK and Treg infiltration rate among tumor types. The high infiltration of NK cells showed significant correlation with patient survival and suggested that immunotherapies which causes the increase of NK cell responses may be efficacious in HNSCC [2].

Regarding the levels of NK receptors and ligands, several have been evaluated in HNSCC studies. NKG2D, which was reported to be downregulated in cervical cancer as described previously, it has been downregulated in HNSCC as well, being accounted for Treg cell activity. NKG2A, in turn, was found upregulated as expected since this receptor induces a negative signalling in NK cell and CD8^+^ T cells through the binding of HLA-E (found overexpressed in cervical cancer [4]). Another study reported this same pattern in HPV-related vulvar intraepithelial neoplasia but instead of be related to poor prognosis, NKG2A was associated with prolonged recurrence-free survival, decreased relapse rate and immunoprotection [122].

As evaluated for cervical cancer, KIR genes and ligands combinations were assessed in HNSCC for their predictive ability for relapse. In a study where it was also evaluated other cancer in addition to HNSCC, it was possible to conclude that the combinations of KIR2DS1/HLAC2C2-C1C2 and KIR3DS1/HLABw4w4-w4w6 showed longer time to treatment (cetuximab) failure (> 10 months) [123]. As KIR/HLA gene system is the main receptor system for regulating NK cell activity and NK cell cytotoxicity is essential for HPV-related cancer resolution, the ability of host KIR genes in expressing suitable KIR for NK cell activation could be an important point for host immune surveillance success.

Furthermore, the KIR receptors and its HLA ligands variability can also affect treatment response in monoclonal antibody therapy, as observed for anti-EGFR therapy in solid tumors. Two combinations of KIR/HLA genotypes showed a better response in this kind of treatment [123]. Therefore, this class of receptors is an interesting study issue, given the wide range of activity and many possible interactions with the highly variable HLA ligands.

### NK cell as therapeutic tool

Regarding the use of NK cells in therapy of cervical and other HPV-related cancers, few alternatives have been tested despite the various immune approaches applied for other cancers [10]. For cervical cancer, allogeneic NK cells from umbilical cord blood or peripheral blood combined with cetuximab caused cervical cancer cells death in vitro independently of HLA-A, -B or -C expression, being suggested as feasible treatment independently of HLA, histology or HPV status of infection [11]. For HNSCC treatment, monalizumab/IPG2201 (anti-NKG2A) is currently under clinical phase I/II for oral carcinoma treatment (http://clinicaltrials.gov NCT02331875) and for metastatic HNSCC, tested with cetuximab (NCT02331875). Another clinical study is currently recruiting for testing lirilumab (anti-KIR monoclonal antibody) in combination with nivolumab and ipilimumab for HNSCC and other solid tumors.

### NKT cell

The natural killer T cell (NKT) is similar to NK cell but displays CD3^+^, CD4^+^ and αβ T cell receptor (TCR) marker repertoire. This cell is able to secrete IL-4 and DC-induced IFN-γ [124], TNF and other cytokines (e.g. IL-10, IL-13) [125]. Its activation occurs through the recognition of CD1d molecules by TCR, and thus, is able to kill tumor cells [126] and induce the maturation of DC that expresses CD40L (activated stage) [127]. CD1d ligands (α-, β-galactosylceramide) have demonstrated protector activity against cancer caused by HPV16 [128] and have been the first to be used therapeutically as adjuvant in DNA vaccine against this viral type [129]. In combination with TLR agonists, these ligands can improve vaccine strategies: the delivering of an HPV16 E7 vaccine together with α-galactosylceramide and MPL, a TLR4 agonist, caused an increase in CTL response, lymphocyte proliferation, IFN-γ synthesis and reduction of tumor volume in a C57BL/6 mice tumor model [130].

HPV has developed mechanisms to avoid NKT cytotoxicity, such as CD1d downregulation [4], but more studies are necessary to elucidate the role of this cell in cancer [131]. It was found that NKT cell induced a paradoxical local immunosuppression in spite of producing IFN-γ [132] and that NKT cells caused immunosuppression and cancer development [133]. Therefore, such cell seems to have dual functions, depending of the lesions stage [12]. A summary of all immune cells studied, and interactions is shown in Table 2.

**Table 2:** Immune cells activities summary

Cell types	Activities										
	Antigen Presentation	Antigen response recall	Phagocytosis	Cytotoxicity	Pro-inflammatory cytokine synthesis	NK cell activation	DC maturation	Antigen-specific response	Antibody-mediated cytotoxicity	Inhibition of proliferation and cell growth	Immunosuppressive activities
Keratinocyte	+	+			+			+			
Stromal cell					+						+
Macrophage	+		+		+						+
Mast cell					+						+
MDSC					+						+
Neutrophil			+	+							+
Dendritic cell	+				+	+		+			+
Langerhans cell	+				+			+			
NK cell	+			+	+		+	+	+	+	

Note. Immune cells have their functions altered by *HPV* infection, playing different roles from those usually performed. Macrophages, for example, can assume two phenotypes with opposite functions depending on HPV interference, *M1* has proinflammatory and antitumor activities while, *M2* is immunomodulatory, promoting tumor cell proliferation and migration, angiogenesis and differentiation to T-regulatory cells [23]

## Conclusions and perspectives

The immune response has a crucial importance in the HPV-related cancer disease progression and resolution. The data highlighted in this review indicate that host immune response may be used to benefit patients through various pathways. These possible new immunological targets break a novel horizon in diagnosis and especially in therapy of cancer. The use of i) cytokines to create the ideal tumor milieu for favouring transformed cells destruction; ii) DC vaccines to induce activation of Th1 and CTLs responses; or iii) activated NK cells by autologous or allogeneic transplants to induce tumoral cells lysis, are very promising immunotherapeutic strategies. Since NK cells are able to kill target cells naturally without MHC restriction and previous sensitization, the use of therapeutic strategies involving NK is very interesting, causing the destruction of cancer cells, even in the presence of the constant immunological modifications undertaken by tumor cells. Also, this review pointed out that the combination of different immunotherapies seems to be crucial to achieve better outcomes as observed in pre-clinical and clinical trials. Promising therapeutic perspectives, thus, are now open for further studies and improvement. Finally, a hypothesis of stromal cell-centered HPV-related carcinogenesis involving immune and HPV-negative dysplastic cells has been drawn by us in the attempt of explaining how HPV-related carcinogenesis may initiate and progress. Also, we suggested that not only cancer-associated fibroblasts play a role in carcinogenesis, but also normal fibroblasts perform pro-tumorigenic functions due to the induction of inflammation, architectural change and immune evasion.  

## Abbreviations

AKt: Protein kinase B
ALDH1: Aldehyde dehydrogenase 1
APC: Antigen presenting cell
Arg-1: Arginase 1
bFGF: basic fibroblast growth factor
CAF: Cancer-associated fibroblast
CCL5/17/22: C-C chemokine ligand 5/17/22
CCR7: C-C chemokine receptor 7
CIN: Cervical intraepithelial neoplasia
COX-2: Ciclo-oxigenase-2
CTL: Cytotoxic T lymphocyte
CTLA-4: Cytotoxic T lymphocyte-associated antigen 4
CXCL9/12: C-X-C chemokine ligand 9/12
CXCR2/4: C-X-C chemokine receptor type 2/4
DC: Dendritic cell
EGF: Epidermal growth factor
EGFR: Epidermal growth factor receptor
EMT: Epithelial-mesenchymal transition
EpCAM: Epithelial cell adhesion molecule
FGFR2b: Fibroblast growth factor receptor 2b
FIGO: International Federation of Gynecology and Obstetrics
Foxp3^+^: Forkhead box P3
Gas-6: Growth arrest-specific 6
GM-CSF/CSF-2: Granulocyte-macrophage colony-stimulating factor
HGF: Hepatocyte growth factor
HLA: Human leukocyte antigen
HLA-DR: Human leukocyte antigen D related
HNSCC: Head and neck squamous cell carcinoma
HPV: Human Papillomavirus
hrHPV: High risk HPV
ICAM: Intercellular adhesion molecule-1
IFN-I/β/γ: (type I interferon and interferon-β or -γ)
IRF: Interferon regulatory factor-1
KC: Keratinocyte
KIR: Killer cell immunoglobulin-like receptor
LC: Langerhans cell
LFA-3: Lymphocyte function-associated antigen 3
LMP-2/7: Large multifunctional proteasome-2/7
LPS: Lipopolysaccharides
MCP-1/CCL2: Monocyte chemoattractant protein-1/C-C chemokine ligand 2
M-CSF/CSF-1: Macrophage colony-stimulating factor/Colony stimulating factor 1
MDSC: Myeloid-derived suppressor cell
MHC: Major Histocompatibility Complex
MICA/MICB: MHC class I polypeptide-related chain A/B
MIP-1α/CCL3: Macrophage inflammatory protein-1α/C-C chemokine ligand 3
MIP-3α/CCL20: Macrophage inflammatory protein-3α/C-C chemokine ligand 20
MMP-7/9: Matrix metalloproteinase 7/9
NF-κB: Nuclear transcription factor kappa B
NK: Natural killer
NLR: Neutrophil:lymphocyte ratio
NO: Nitric oxide
OPSCC: Oropharyngeal squamous cell carcinoma
OSCC: Oral squamous cell carcinoma
PAMPs: Pathogen-associated molecular patterns
PD-1: Programmed death-1 receptor
PDGFRα: Platelet derived growth factor receptor α
PD-L1: Programmed death ligand-1
PI3K: Phosphoinositide 3-kinase
Poly(I:C): Polyriboinosinic-polyribocytidylic acid
SOX2: Sex determining region Y
TAM: Tumor-associated macrophage
TAN: Tumor-associated neutrophil
TAP-1/2: Transporter antigen processing-1/2
TGF-β: Transforming growth factor β
TIM-3: T cell immunoglobulin mucin 3
TLR: Toll-like receptor
TNF-α/β: Tumor necrosis factor α and tumor necrosis factor β
Treg: T-regulatory lymphocyte (CD4^+^ CD25^+^ Foxp3^+^)
VEGF: Vascular Endothelial Growth Factor
VLP: Virus-like particle
